# Selection of a *Rhizobium* sp. Strain and Culture Medium for the Development of a Liquid Bioinoculant for Rice (*Oryza sativa* L.) Cultivar Selección 1

**DOI:** 10.3390/microorganisms14050998

**Published:** 2026-04-29

**Authors:** Claudia Pérez-Arabi, Ionel Hernández-Forte, Lisbel Travieso-Hernández, María C. Nápoles-García, Vivianne Machado-Brito, Belkis Morales-Mena, Kevin Verdugo-Chavez, María José Villarroel-Contreras, Héctor Herrera

**Affiliations:** 1 Plant Physiology and Biochemistry Department, National Institute of Agricultural Science, Carretera a Tapaste Km 3 y ½, San José de las Lajas 32700, Mayabeque, Cuba; claudia.arabi94@gmail.com (C.P.-A.); lisbeltravieso@gmail.com (L.T.-H.); mariacaridad.napoles@gmail.com (M.C.N.-G.); vivi.machado2899@gmail.com (V.M.-B.); bmorales@inca.edu.cu (B.M.-M.); 2 Microbial Biochemistry and Genomics Department, Clemente Estable Biological Research Institute, Montevideo 11600, Uruguay; 3 Center for Biodiversity and Ecological Sustainability (C-BEST), Facultad de Ciencias Agropecuarias y Medioambiente, Universidad de La Frontera, Temuco 4811230, Chile; k.verdugo02@ufromail.cl (K.V.-C.); mjvillarroelc_@outlook.com (M.J.V.-C.)

**Keywords:** plant growth-promoting bacteria, indole-3-acetic acid production, bacterial motility, antifungal activity, inoculant formulation stability

## Abstract

Plant growth-promoting bacteria (PGPB)-based inoculants represent a promising alternative to mineral fertilizers. However, their application may be limited by constraints associated with the use of living microorganisms, particularly under field conditions. The objective of this study was to select a bacterial strain and a suitable carrier for the inoculation of rice cv. Selección 1. The effect of inoculation with 3 *Rhizobium* spp. strains on rice growth was evaluated under greenhouse conditions, enabling selection of the most promising strain. This strain was further characterized based on its motility, production of indolic compounds in the presence of tryptophan, and antagonistic activity against 3 rice phytopathogenic fungi. In addition, the effects of culture media based on aqueous extracts of soybean and rice seeds on bacterial growth and chemotactic response were evaluated, along with the shelf-life stability of the resulting inoculant formulations. *Rhizobium* sp. strain 5P1 significantly increased plant height (33%), root length (21%), shoot dry weight (30%), and root dry weight (17%) of rice cultivar *Selección 1* under greenhouse conditions. The strain exhibited motility predominantly via swarming and twitching, produced indolic compounds (23.9 ± 0.8 µg mL^−1^), and showed antagonistic activity against *Magnaporthe oryzae* (32.5% radial growth inhibition at 16 days), *Curvularia oryzae* (20.0%), and *Bipolaris oryzae* (6.6%) under in vitro conditions. Culture media based on molasses and soybean or rice seed extracts did not enhance bacterial growth relative to the conventional medium; however, they elicited a stronger chemotactic response. Formulations supplemented with sodium alginate and carboxymethylcellulose maintained cell viability above 10^8^ CFU mL^−1^ after 105 days of storage at 4 °C. These findings propose *Rhizobium* sp. strain 5P1 and a molasses-based carrier formulation as strong candidates for the development of an effective bioinoculant for rice in Ferric Gleysol soils.

## 1. Introduction

The use of chemical and mineral fertilizers has been a fundamental pillar of the sustained increase in global agricultural productivity, as they supply essential nutrients that correct soil deficiencies and maintain adequate nutrient balances for crop development [1,2]. However, their production and application have generated significant environmental impacts, including surface and groundwater contamination, eutrophication of aquatic ecosystems, greenhouse gas emissions, and progressive soil quality degradation [3,4]. In this context, inoculants based on plant growth-promoting bacteria (PGPB) have gained increasing relevance as a partial or complementary alternative to conventional mineral fertilization [5,6]. These bioproducts have been shown to enhance yield and nutrient use efficiency across a wide range of crops, including cereals, legumes, vegetables, and fruit crops, particularly when integrated into appropriate fertilization schemes [7,8].

PGPB promote plant growth through a range of mechanisms generally classified as either direct or indirect, depending on how the growth-promoting effect is exerted. The most important direct mechanisms include biological nitrogen fixation (BNF), inorganic phosphorus solubilization, and siderophore production, as well as the synthesis of phytohormones and plant growth regulators, such as auxins, gibberellins, cytokinins, and abscisic acid, which directly influence plant development [9,10]. Indirect mechanisms involve antagonistic activity against plant pathogens, thereby reducing their populations and, consequently, their detrimental effects on plants. These mechanisms include the production of allelochemical compounds (e.g., antibiotics, hydrogen cyanide [HCN], aldehydes, alcohols, and ketones), the secretion of lytic enzymes, and the induction of systemic resistance responses [11,12]. Among the most representative PGPB genera are *Azospirillum*, *Bacillus*, *Pseudomonas*, *Arthrobacter*, *Enterobacter*, *Klebsiella*, *Serratia*, *Burkholderia*, *Paenibacillus*, *Streptomyces*, and *Microbacterium*, along with bacteria belonging to the rhizobial group [13,14]. Rhizobia have been traditionally studied for their ability to establish highly specialized symbiotic associations with leguminous plants [15,16]. However, growing attention has been directed toward their capacity to act as PGPB in non-leguminous plants, including economically important grasses such as *Oryza sativa* (rice), *Sorghum bicolor* (sorghum), and *Zea mays* (maize) [17,18,19,20]. In rice specifically, accumulating evidence supports the role of rhizobia as rhizospheric and endophytic colonizers capable of promoting the growth of this cereal crop [21,22].

From a biotechnological production standpoint, PGPB-based inoculants are final formulated products comprising a carrier and either a single bacterial strain or a consortium of microorganisms. The term ‘carrier’ refers to an abiotic substrate (solid, liquid, or gel) used during the formulation process, which involves the integration of the selected bacterial strain into the carrier at the laboratory or industrial scale [23]. Despite the agronomic benefits of these inoculants, they present several limitations that constrain their biotechnological potential, most notably the rapid decline of bacterial populations during storage. Bacterial viability is adversely affected by exposure of cellular macromolecules to toxic oxygen levels and elevated temperatures during storage [24]. A minimum cell density threshold is required to elicit the desired plant response; for example, rhizobial inoculants typically require 10^6^–10^9^ cells per plant [25,26]. Therefore, both formulation and storage conditions must provide a suitable microenvironment that prevents rapid loss of cell viability and ensures the availability of viable cells in the rhizosphere, where they can effectively interact with plants and the soil microbiome [23].

The use of low storage temperatures (4–6 °C), together with specific additives or supplements during formulation, reduces water and thermal stress, thereby maintaining cell viability and inoculant effectiveness throughout storage [27]. In addition, polymers such as carboxymethylcellulose (CMC), polyvinylpyrrolidone, and sodium alginate have been widely used as formulation additives owing to their low cost and compatibility with microorganisms [28,29]. Furthermore, salts such as calcium chloride (CaCl_2_), particularly when combined with alginate, have also been employed for this purpose, as they enable the formation of calcium–alginate matrices that protect bacterial cells and enhance their survival during storage [30]. No universal formulation exists that satisfies all the physiological requirements of microorganisms, not even for rhizobia, which are among the most extensively studied and widely used bacteria in commercial agricultural inoculants. Therefore, selecting an appropriate formulation is a case-specific process and is as critical as the selection of the bacterial strain itself for successful crop inoculation.

In Cuba, a culture medium known as Bradyfact^®^ was developed and optimized over more than 24 years. This medium, composed of mineral salts, molasses, and an aqueous soybean extract, serves as the carrier of the Cuban biofertilizer Azofert^®^. Bradyfact^®^ enhances the multiplication of *Rhizobium leguminosarum* and *Bradyrhizobium elkanii* strains and induces Nod factor synthesis to a greater extent than conventional commercial media such as yeast extract-mannitol (YM) medium [31,32,33]. Application of these inoculants to *Glycine max* L. (soybean), *Phaseolus vulgaris* (common bean), and *Canavalia ensiformis* (jack bean) increases effective nodulation, biological nitrogen fixation (BNF), biomass accumulation, and agricultural yield [33,34,35,36]. However, no inoculants based on this technology are currently available in Cuba for economically important cereal crops such as rice.

Rice is a prioritized crop in Cuba, with an annual per capita consumption of approximately 70 kg, one of the highest in Latin America. However, national production, estimated at approximately 226,000 tons, does not meet domestic demand [37,38]. In particular, the Cuban rice cv. Selección 1 is one of eleven cultivars designated for cultivation in the country’s main rice-producing regions, owing to its high yield potential under national production conditions (5.8–7.1 t ha^−1^) and excellent milling quality [39]. Furthermore, its cultivation has expanded considerably in recent years, with planted areas reaching up to 47,000 ha. Given the environmental and economic impacts associated with the expansion of intensive cropping systems such as rice, the development of more environmentally sustainable alternatives, such as PGPB-based inoculants, including those derived from rhizobia, is essential. In this context, studies on PGPB in Cuba have focused on the isolation, characterization, and application of native rhizospheric and endophytic bacteria, mainly strains belonging to genera such as *Pseudomonas* and *Burkholderia*, demonstrating their capacity to enhance rice growth through mechanisms including phytohormone production, biological nitrogen fixation, phosphate solubilization, and antagonism against phytopathogens [40,41,42]. More recent studies have reported *Pantoea*, *Acinetobacter*, and *Mitsuaria* as genera associated with and promoting the growth of the Cuban rice cv. INCA LP-7 [43]. The main advances in the rhizobia–rice interaction in Cuba have been achieved at the National Institute of Agricultural Sciences (INCA) using the Cuban cultivars INCA LP-5 and INCA LP-7. A well-characterized collection of rhizobial strains was obtained from these cultivars, and the ability of selected strains to promote rice growth and nutrition has been demonstrated [44,45,46,47,48,49]. However, this capacity has only been confirmed in the cultivars from which the strains were originally isolated, limiting their potential application to other widely cultivated varieties in the country, such as *Selección 1*. To date, only one study conducted in Cuba has evaluated the effect of PGPB on the rice cv. Selección 1, reporting that the bio-input Bioeraiz^®^ promotes plant growth [50]. However, several aspects remain to be clarified. The bacterial strain used in that study does not originate from rice and may not be adapted to the environmental conditions under which this crop is cultivated in Cuba [50]. In addition, the growth-promoting effect was attributed to the PGPB-free fraction of the product, obtained after removal of bacterial cells by centrifugation during the final stage of the production process, which may increase production costs compared to formulations containing viable PGPB. Furthermore, the assays were conducted using Ferralitic Red soil, which is not representative of the main rice-growing areas in western Cuba, potentially limiting the extrapolation of these findings to field conditions. Therefore, actually no rhizobia-based inoculants are available for rice. Given the agronomic importance of this cultivar, standardizing the use of bacterial strains with the potential to be incorporated into current production systems is essential. Therefore, this study aimed to select a bacterial strain and a suitable carrier for rice biofertilization in Ferric Gleysol soils.

## 2. Materials and Methods

### 2.1. Biological Material

A total of three *Rhizobium* spp. strains were used in this study, all of which are part of the collection of the Department of Plant Physiology and Biochemistry at the National Institute of Agricultural Sciences (INCA) (Table 1). Additionally, 3 pathogenic strains directly isolated from infected rice plants were selected for antagonistic activity assays (Table 1). All strains had been previously characterized as PGPB and are considered promising candidates for rice inoculation [46,47,48,49,51]. Strains were cultured in Tryptone-Yeast (TY) medium (per liter: 5 g tryptone, 3 g yeast extract, 0.68 g CaCl_2_) at 150 rpm in an orbital shaker (HDL Apparatus, HZQ-F200, ASLi Test Equipment Co., Ltd., Dongguan, China) for 16 h at 30 °C.

Certified seeds of the rice cv. Selección 1, provided by the Los Palacios Experimental Station of the National Institute of Agricultural Sciences (Cuba), were used. Seeds were pre-germinated in Petri dishes containing filter paper moistened with 10 mL of sterile distilled water. A total of 25 seeds were placed per dish and incubated in the dark at 28 °C for 72 h.

### 2.2. Substrate Chemical Analysis

A substrate was prepared for rhizobial inoculation assays using rice plants (cv.r Selección 1). The substrate consisted of a 3:1 (*v*/*v*) mixture of Ferric Gleysol soil and organic matter. Soil was collected from the Los Palacios Experimental Station, National Institute of Agricultural Sciences (Cuba) (22°33′59″ N, 83°14′15″ W; 32 m a.s.l.). This soil type is representative of the main rice-growing areas of western Cuba and is characterized by low natural fertility and imperfect drainage, conditions typical of irrigated rice production systems [47].

Substrate samples (500 g) were characterized according to the methods described by González-Fernández et al. [52]. Briefly, substrate pH was measured potentiometrically in substrate:KCl suspensions (1:2.5, *w*/*v*). Organic matter content (OM, %) was determined using the Walkley–Black method. Available P_2_O_5_ (mg 100 g^−1^ soil) was extracted using 0.1 N sulfuric acid at a substrate-to-solution ratio of 1:2.5 for 3 min; phosphorus was then determined colorimetrically. Exchangeable cations were extracted with 1 mol L^−1^ NH_4_OAc and quantified by complexometric titration (Ca^2+^ and Mg^2+^) and flame photometry (Na^+^ and K^+^).

### 2.3. Effect of Rhizobium spp. Inoculation in Rice Growth Under Greenhouse Condition

Pre-germinated rice seeds were sown in pots containing 0.27 kg of the non-sterilized substrate described above. A total of 3 seedlings were placed per pot. Each rice seedling was then inoculated with 300 μL of bacterial suspension (5 × 10^9^ CFU mL^−1^) cultured in TY medium, applied directly onto the seedling. Uninoculated plants served as the negative control. A total of 10 pots were used per treatment in a completely randomized design. Each pot was placed in a tray containing a diluted Hoagland nutrient solution (1:2 *v*/*v*), which supplied the nutrients required for plant growth within the limited soil volume. Plants were grown under greenhouse conditions with a 16 h light/8 h dark photoperiod at 26/22 °C (day/night) and 70% relative humidity.

At 15 days post-inoculation (DPI), 1 seedling was removed per pot, leaving 2 plants per pot. At 30 DPI, relative total chlorophyll content (SPAD units) was measured in 2 leaves per plant, including the flag leaf, using a portable chlorophyll meter (SPAD-502, Konica Minolta, Ramsey, NJ, USA). At 50 DPI, plant height (cm), root length (cm), shoot dry weight (g), and root dry weight (g) were determined using an analytical balance (DENVER SI-602, Passau, Germany). The most promising strain was then selected for further characterization of PGP traits.

### 2.4. Characterization of Selected Strain for PGP Traits

#### 2.4.1. Motility Assays

The bacterial strain was grown in liquid TY medium and the cell concentration adjusted to OD_600_ = 1.0. A total of 5 microliters of the inoculum were spotted in triplicate onto compartmentalized Petri dishes containing TY medium solidified with different agar concentrations, 0.1% (*w*/*v*), 0.5% (*w*/*v*), and 1.0% (*w*/*v*), to assess swimming, swarming, and twitching motility, respectively. Sterile TY medium was spotted under identical conditions as a negative control. Plates were incubated at 30 °C for 48 h. Motility was assessed by measuring the diameter of the bacterial spreading zone at 0, 24, and 48 h post-inoculation, from the point of inoculation to the colony edge.

#### 2.4.2. Indolic Compound Production

Indolic compound production was detected using a colorimetric method in TY medium supplemented with tryptophan (200 µg mL^−1^) as a biosynthetic precursor. After 72 h of growth at 30 °C, samples were centrifuged at 12,000× *g* for 5 min, and 150 µL of each supernatant was incubated for 30 min in the dark with 100 µL of Salkowski reagent. Indolic compound concentration was determined colorimetrically by measuring absorbance at 540 nm [53]. Development of a pink coloration was interpreted as a positive result. *Gluconacetobacter diazotrophicus* Pal5 was used as a positive control. The assay was performed in duplicate with 3 replicates per run.

#### 2.4.3. Antagonistic Activity Against Fungal Phytopathogens

The antagonistic activity of the selected isolate against 3 rice phytopathogens (*Magnaporthe oryzae* strain 28, *Curvularia oryzae* strain 1, and *Bipolaris oryzae* strain 1, Micoteca, UCTB-LP, INCA) was evaluated using a dual culture assay on PDA medium, as previously described [48]. Fungal plugs were placed at the center of the plates, and 10 μL of the selected isolate suspension was streaked along the plate border. Plates were incubated in the dark at 30 °C, and the percentage of radial growth inhibition (PRGI) was recorded at 16 days for *M. oryzae* and at 4 days for *C. oryzae* and *B. oryzae* [54]. All assays were performed in triplicate.

### 2.5. Evaluation of Alternative Culture Media for the Selected Strain

#### 2.5.1. Bacterial Growth

The bacterial strain was grown in four culture media for 16 h at 150 rpm in an orbital shaker (HDL Apparatus, HZQ-F200, ASLi Test Equipment Co., Ltd., Dongguan, China) at 30 °C: TY medium, modified TY medium (pH 5.0, 1.5% NaCl) (TYm), and two modified versions of Bradyfact^®^ medium containing molasses as the carbon source [55]. In the first modified Bradyfact^®^ medium, pH was adjusted to 5.0 and NaCl concentration to 1.5%, while the remaining components were kept unchanged (Bf-Sm). In the second, the soybean aqueous extract was replaced with an equivalent volume of rice seed aqueous extract (Ers2V1). Five replicates were used per culture medium. Bacterial concentration in each medium was determined at the end of the experiment by the decimal serial dilution method, with dilutions plated onto TY medium and incubated at 30 °C for 48 h.

#### 2.5.2. Chemotactic Response

A chemotaxis assay was performed to determine the ability of the selected strain to detect and respond directionally to compounds present in the culture media. The rhizobial strain was grown in 5 mL of TY medium for 24 h and the cell concentration adjusted to 5 × 10^9^ CFU mL^−1^. The culture was centrifuged at 3000 rpm for 3 min, the supernatant discarded, and the cell pellet resuspended in sterile saline solution (0.9% NaCl). This washing step was repeated twice to remove residual TY medium. In parallel, pulled glass capillaries (10 cm length, ~5 µm tip diameter) were filled in triplicate with each culture medium and immersed in the cell suspension for 1 h. The negative control consisted of identical capillaries filled with sterile saline solution. The capillary contents were then expelled, and bacterial cell concentration in the recovered suspensions was determined by the decimal serial dilution method, followed by plating on TY medium and incubation at 28 °C for 48 h. Culture media yielding the highest bacterial concentrations and eliciting a chemotactic response were selected to evaluate the effect of the inoculant on rice growth and physiology under reduced fertilization conditions. The medium eliciting the strongest chemotactic response was subsequently selected to evaluate the shelf-life stability of the strain.

### 2.6. Effect of Culture Medium Formulations on the Shelf-Life Stability of the Strain

A stepwise scale-up process of the selected strain and culture medium was carried out, maintaining a constant inoculation ratio of 10% (*v*/*v*) at each stage. A pre-inoculum (25 mL) was used to inoculate 250 mL of the same medium, which was subsequently used to inoculate 2500 mL. All cultures were grown in Erlenmeyer flasks at a working volume corresponding to 20% of the total flask capacity to ensure adequate aeration and incubated at 150 rpm in an orbital shaker (HDL Apparatus, HZQ-F200) at 30 °C for 16 h at each stage. The final inoculant reached a concentration of 1 × 10^10^ CFU mL^−1^. In parallel, the same sterile culture medium was prepared and supplemented with different chemical compounds reported as potential preservatives: CaCl_2_ (20 g L^−1^), sodium alginate (20 g L^−1^), and CMC (2 g L^−1^). Formulations were then obtained by mixing the bacterial inoculant with each culture medium containing the respective preservative (1:1, *v*/*v*), followed by thorough homogenization and redistribution into 200 mL flasks. Flasks were stored at 4 °C for 105 days. Every 21 days, bacterial concentration was determined in 3 randomly selected flasks per treatment using the decimal serial dilution method, with dilutions plated on YM medium and incubated at 28 °C for 72 h.

### 2.7. Statistical Analysis

Data from the greenhouse inoculation assays, motility assay, indolic compound production, antagonistic activity, bacterial concentration in different culture media, and chemotaxis assays were subjected to tests of normality (Kolmogorov–Smirnov test) and homogeneity of variance (Bartlett’s test). A one-way analysis of variance (ANOVA) followed by Tukey’s HSD post hoc test was applied for mean comparisons at *p* ≤ 0.05. Statistical analyses were performed using Statgraphics Centurion XVI (Statpoint Technologies, Inc., Warrenton, VA, USA), and data were visualized using Microsoft^®^ Excel^®^ for Microsoft 365 MSO (v. 2508).

## 3. Results

The substrate had a pH of 6.57 ± 0.27 (KCl), an organic matter content of 3.05 ± 0.70%, and an available phosphorus concentration of 75.1 ± 6.5 mg 100 g^−1^ soil. Exchangeable cation concentrations were 11.62 ± 0.97 cmolc kg^−1^ for Ca^2+^, 4.75 ± 0.1 cmolc kg^−1^ for Mg^2+^, trace levels of Na^+^, and 0.80 ± 0.02 cmolc kg^−1^ for K^+^ (Table 2). These physicochemical properties fall within ranges reported for soils used in rice cultivation [39].

### 3.1. Rhizobium sp. Strain 5P1 Promotes the Growth of Rice cv. r *Selección 1* Under Greenhouse Condition

**Figure 1:**
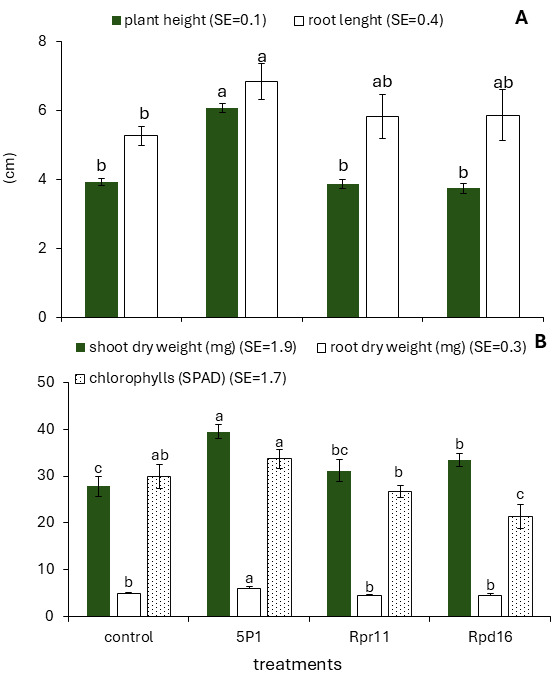
Effect of inoculation with strains 5P1, Rpr11, and Rpd16 on plant height and root length (**A**), shoot dry weight and root dry weight (**B**), at 50 days post-inoculation (DPI), and relative chlorophyll content (SPAD units) at 30 DPI (**B**) of rice plants cv. *Selección 1* under greenhouse conditions. The control treatment consisted of uninoculated plants. Data are presented as means ± standard error (SE); different letters above bars indicate statistically significant differences among treatments (Tukey’s HSD, *p* ≤ 0.05, *n* = 20).

*Rhizobium* sp. strain 5P1, isolated from the rhizosphere of rice cv. INCA LP-7, promoted the growth of rice cv. Selección 1 at 50 days after inoculation (DAI) under greenhouse conditions, as evidenced by significant increases in plant height, root length, shoot dry weight, and root dry weight (Figure 1). No significant differences were observed between control plants and those inoculated with strains Rpr11 and Rpd16 in plant height, root length, or root dry weight (Figure 1A,B). Particularly, strain Rpd16 increased shoot dry weight relative to the control (Figure 1B). In contrast, plants inoculated with strain 5P1 exhibited the highest relative chlorophyll content among all treatments; however, this value did not differ significantly from that of the uninoculated control (Figure 1B). Collectively, these results indicate that *Rhizobium* sp. strain 5P1 was the most promising strain under greenhouse conditions and was therefore selected for subsequent experiments.

### 3.2. Rhizobium sp. Strain 5P1 Exhibits Plant Growth-Promoting Traits

**Figure 2:**
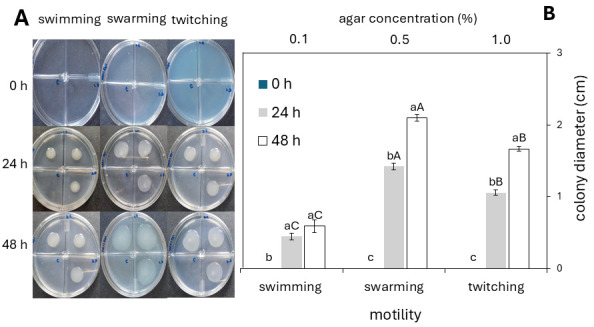
Motility of *Rhizobium* sp. strain 5P1 on TY medium solidified with 0.1% (*w*/*v*), 0.5% (*w*/*v*), and 1.0% (*w*/*v*) agar to assess swimming, swarming, and twitching motility, respectively, at 0, 24, and 48 h at 30 °C. (**A**) In each compartmentalized Petri dish, 5 µL of *Rhizobium* sp. strain 5P1 inoculum (OD_600_ = 1.0) was spotted in triplicate. The negative control consisted of 5 µL of sterile TY medium spotted in the lower-left compartment of each Petri dish. (**B**) Data are presented as means ± standard error (SE) of 9 replicates (Tukey’s HSD, *p* ≤ 0.05, *n* = 9). Lowercase letters indicate significant differences among evaluation time points (0, 24, and 48 h); uppercase letters indicate significant differences among motility types (swimming, swarming, and twitching); means sharing the same letter do not differ significantly.

*Rhizobium* sp. strain 5P1 exhibited all 3 motility types (swimming, swarming, and twitching) on TY medium after 48 h of incubation (Figure 2A). Significant differences in colony diameter were observed between 24 and 48 h for swarming and twitching motility, whereas no significant differences were detected for swimming motility over the same period. Swarming produced the largest colony diameters, followed by twitching, while swimming yielded the smallest diameters at both time points (Figure 2B).

**Figure 3:**
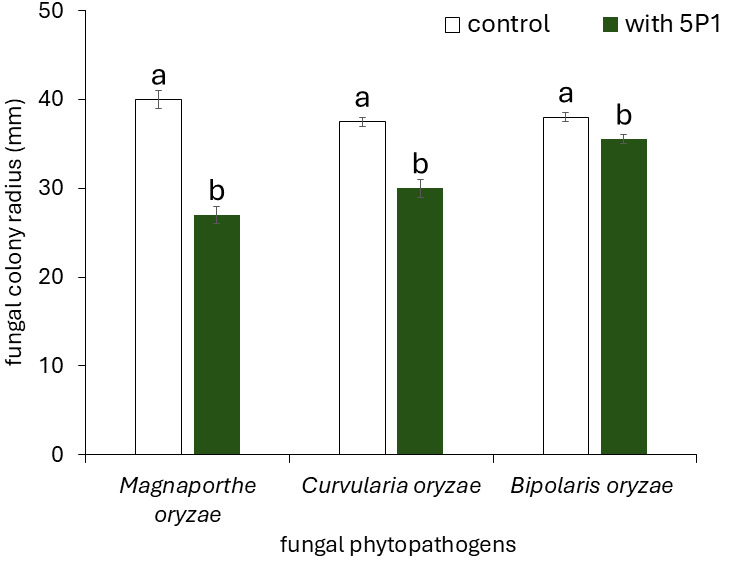
Antagonistic activity of *Rhizobium* sp. strain 5P1 against the fungal phytopathogens *Magnaporthe oryzae* strain 28 (evaluated at 16 days), *Curvularia oryzae* strain 1, and *Bipolaris oryzae* strain 1 (evaluated at 4 days) in dual-culture assays on PDA medium. Data are expressed as mean radial growth (cm) of each fungus in the negative control and in confrontation with *Rhizobium* sp. strain 5P1. Values represent means ± standard error of 3 replicates (Student’s *t*-test, *p* ≤ 0.05, *n* = 3).

*Rhizobium* sp. strain 5P1 produced 23.9 ± 0.8 µg mL^−1^ of indolic compounds in TY medium supplemented with tryptophan. The antagonistic activity of the strain was further evaluated against three rice fungal pathogens: *M. oryzae*, *C. oryzae*, and *B. oryzae*. The strain inhibited the radial growth of all three pathogens (Figure 3). Radial growth of *M. oryzae* was reduced by 32.5% at 16 DPI, while *C. oryzae* and *B. oryzae* were inhibited by 20.0% and 6.6%, respectively, after 4 days of exposure.

### 3.3. High Bacterial Growth of Rhizobium sp. Strain 5P1 Across Alternative Culture Media

The results showed high concentrations of *Rhizobium* sp. strain 5P1 in all tested culture media, ranging from 10^9^ to 10^10^ CFU mL^−1^, with no significant differences among treatments (Table 3). Based on these results, the TYm, Bf-Sm, and Ers2V1 media were selected for the chemotaxis assay.

### 3.4. Alternative Culture Media Enhance Chemotaxis in Rhizobium sp. Strain 5P1

The chemotaxis assay revealed that modified Bradyfact^®^ media supplemented with aqueous seed extracts (soybean and rice) elicited the highest cell accumulation of *Rhizobium* sp. strain 5P1. No significant differences in bacterial concentration were observed between modified TYm medium and the saline solution negative control. The highest cell concentration (2.8 × 10^6^ CFU mL^−1^) was recorded in the modified Bradyfact^®^ medium supplemented with soybean seed extract (Bf-Sm) (Table 3). This medium was therefore selected to evaluate the shelf-life stability of the strain in formulations stored at 4 °C.

### 3.5. Formulations Improve the Shelf-Life Stability of Rhizobium sp. Strain 5P1

**Figure 4:**
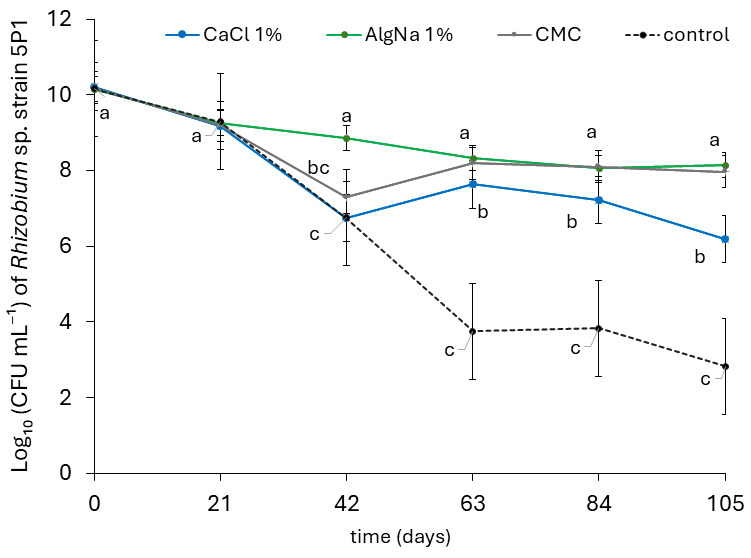
Concentration of *Rhizobium* sp. strain 5P1 in formulated inoculants stored at 4 °C for 105 days. Formulations were prepared by mixing bacterial cultures grown for 16 h in modified Bradyfact^®^ medium (Bf-Sm) with the same sterile medium (1:1, *v*/*v*), supplemented with CaCl_2_ (20 g L^−1^), sodium alginate (20 g L^−1^), or carboxymethylcellulose (CMC) (2 g L^−1^). Bacterial concentration was assessed every 21 days up to 105 days. The control consisted of the unsupplemented formulation. Data are presented as means ± standard error of 3 replicates per sampling point (Tukey’s HSD, *p* ≤ 0.05, *n* = 3). Different letters above data points indicate significant differences among formulations at each time point.

Bacterial concentration of *Rhizobium* sp. strain 5P1 declined progressively throughout the storage period, beginning at the first evaluation point at 21 days. Nevertheless, from day 42 onward, the formulation supplemented with sodium alginate, and from day 63 onward, those supplemented with CaCl_2_ and CMC, maintained higher bacterial concentrations than the unsupplemented control (Figure 4). Formulations containing sodium alginate and CMC sustained the highest bacterial concentrations (on the order of 10^8^ CFU mL^−1^) after 105 days of storage at 4 °C.

## 4. Discussion

Over the past several years, our research has focused on fundamental studies involving the isolation, identification, and characterization of bacteria associated with the rhizosphere and seeds of rice and maize [45,48,56,57]. However, it remains unclear whether similar effects occur in other rice cultivars of agronomic relevance in Cuba, such as cv. Selección 1. This cultivar is characterized by high yield potential and disease resistance, making it particularly attractive for cultivation. Moreover, no previous studies have addressed the interaction between this cultivar and PGPB; therefore, the present work contributes to a better understanding of rhizobial strain–rice cultivar specificity.

Inoculation of rice plants with *Rhizobium* sp. strain 5P1 under greenhouse conditions resulted in a significant stimulatory effect on plant growth (Figure 1). The growth-promoting effects of rhizobia and other PGPB on the biomass of gramineous crops have been widely reported [58,59,60]. For instance, rice plants inoculated with *Rhizobium leguminosarum* and *Bradyrhizobium* sp. exhibited increases of 20% and 18% in total biomass, respectively [61,62]. These growth-stimulatory effects are commonly attributed to the ability of rhizobial strains to produce phytohormones such as indole-3-acetic acid (IAA) [63], which influence plant development by increasing plant height, leaf elongation, shoot biomass, and tiller formation [64]. Consistent with these observations, previous studies have shown that inoculation with endophytic *Sinorhizobium meliloti* isolated from rice activates auxin-related and cell division pathways, leading to increased shoot height and biomass accumulation [19].

Although *Rhizobium* sp. strain 5P1 and *Rhizobium* sp. strain Rpd16 were originally isolated from rice cultivars INCA LP-5 and INCA LP-7, respectively, both effectively promoted growth in rice cv. Selección 1, indicating a low degree of host specificity. In non-leguminous plants, rhizobia primarily function as PGPB through general mechanisms of biofertilization, phytostimulation, and biocontrol. Previous studies have reported similar findings. For example, *Rhizobium leguminosarum* bv. *trifolii* SN10, isolated from root nodules of *Trifolium alexandrinum* L., was shown to promote the growth of 4 different rice varieties while successfully colonizing the root surface [65]. Likewise, rhizobia isolated from nodules of *Glycine max* (soybean) and *Desmodium incanum* increased plant height and biomass in *Avena strigosa*, *Paspalum notatum*, *Urochloa decumbens*, and *Lolium multiflorum* [58,66]. The capacity of a single rhizobial strain to promote growth across different rice cultivars, as observed in this study, represents a clear advantage for the development of broadly applicable bioinoculants across diverse cropping systems.

Based on the greenhouse results, *Rhizobium* sp. strain 5P1 was selected and partially characterized with the aim of identifying potential mechanisms involved in rice colonization and growth promotion This preliminary characterization could provide information on the motility capacity of the bacteria under study and offer insights into the possible mechanisms of root colonization. Swarming and twitching were the predominant motility types exhibited by this strain (Figure 2). Mutants of *Rhizobium leguminosarum* and *Sinorhizobium meliloti* unable to synthesize surface lipopolysaccharides (LPS), exopolysaccharides (EPS), flagella, or type IV pili have been shown to exhibit impaired or abolished swarming and twitching motility, highlighting the contribution of these structures to bacterial motility [67,68]. A hydrated cell surface mediated by EPS, together with peritrichous or subpolar flagella, promotes swarming motility by enabling rapid surface translocation; whereas twitching motility, mediated by type IV pili, involves slower, discontinuous, short-range cell movements [69,70]. This mechanistic difference may account for the larger colony diameters consistently observed during swarming compared to twitching and swimming motility.

*Rhizobium* sp. strain 5P1 demonstrated the ability to produce indolic compounds in the presence of tryptophan as a precursor. Among these compounds, IAA is most commonly associated with phytostimulatory mechanisms in PGPB [71,72]. Plant root exudates, including those of rice, contain several amino acids, among them tryptophan, which acts as a biosynthetic precursor of indolic compounds in the rhizosphere [73,74]. The combined capacity of strain 5P1 to deploy multiple motility strategies and synthesize indolic compounds likely underpins its ability to efficiently colonize rice roots and promote plant growth.

*Rhizobium* sp. strain 5P1 demonstrated antagonistic activity against *M. oryzae*, *C. oryzae*, and *B. oryzae* under *in vitro* conditions, significantly inhibiting the radial growth of all three phytopathogens (Figure 3). *M. oryzae*, the causal agent of rice blast, can infect all aerial parts of the plant at advanced disease stages, resulting in yield losses exceeding 50% [75,76]. Although less impactful than *M. oryzae*, both *B. oryzae* and *C. oryzae* are recognized as important rice pathogens in Cuba, causing significant yield reductions and increasing production costs [77,78,79]. *B. oryzae* causes brown spot disease, affecting leaves, seedlings, stems, sheaths, and grains. In contrast, *C. oryzae* causes leaf spot, seedling rot, and grain discoloration, typically acting as a secondary pathogen [80,81]. The antagonistic activity exhibited by this rhizobial strain represents a valuable plant growth-promoting trait, particularly for incorporation into an agricultural bioproduct. However, in vivo assays are required to confirm its biocontrol effectiveness under field-relevant conditions. Several PGPB genera, including *Pseudomonas*, *Bacillus*, and *Enterobacter*, have demonstrated marked inhibitory effects against *Curvularia* spp. under *in vitro* conditions and, in some cases, reduced disease severity caused by *Curvularia lunata* in rice under greenhouse conditions [82,83]. Although the *in vitro* antagonistic activity of *Rhizobium* sp. strain 5P1 against rice phytopathogens is promising, its effectiveness under field conditions remains to be confirmed. This is particularly relevant in Ferric Gleysol soils, which are characterized by high moisture content, periodic water saturation, reduced oxygen availability, and fluctuating redox conditions [84,85,86], all of which may affect the survival and activity of introduced inoculants. Furthermore, competition with native soil microbiota may additionally constrain their functional efficacy under real conditions. However, *Rhizobium* sp. strain 5P1 was isolated from Ferric Gleysol soil associated with the rhizosphere of rice cultivated under production conditions and is therefore adapted to the prevailing edaphoclimatic conditions of these systems [47].

We further evaluated the effect of two alternative culture media and formulations on bacterial growth, chemotactic response, and shelf-life of *Rhizobium* sp. strain 5P1, with the aim of identifying a promising carrier for this strain. The relatively high cell concentrations (10^9^–10^10^ CFU mL^−1^) obtained in both alternative media (Table 3) suggest that conventional commercial media such as YM medium could be partially or fully replaced by these formulations. Culture media are key factors influencing both the population size and physiological status of microorganisms in inoculants, ultimately affecting their performance under field conditions [87]. Several studies have shown that culture media formulated from agro-industrial by-products can support high bacterial biomass yields comparable to, or even exceeding, those obtained with commercial media, while significantly reducing production costs. Bashan, de-Bashan, Prabhu and Hernandez [23] highlighted that the use of agro-industrial by-products, such as molasses, corn steep liquor, and dairy residues, as raw materials for culture media represents a viable strategy to replace traditional laboratory media, enabling high bacterial biomass production while substantially reducing costs associated with large-scale inoculant manufacture. Similarly, culture media prepared from plant-based feedstocks combined with agro-industrial by-products such as molasses and glycerol have been shown to support *Rhizobium leguminosarum* growth with higher dry biomass yields and shorter generation times than conventional YM medium [88].

Our results also demonstrated that the alternative media elicited chemotactic response in strain 5P1 (Table 3). The ability of this strain to move via swimming and twitching, and predominantly through swarming (Figure 2), together with the chemotactic mechanisms triggered by chemical signals present in these media, may account for this behavior. Molasses, a component common to both alternative media evaluated, is a sugar industry by-product characterized by a complex and heterogeneous chemical composition, including fermentable carbohydrates, free amino acids, organic nitrogen, essential minerals, vitamins, and secondary organic compounds that microorganisms can exploit not only as nutrient sources but also as signaling molecules [89]. Furthermore, seeds release a wide variety of soluble metabolites into the soil that serve as nutrients and act as chemoattractants and signaling compounds for soil bacteria, triggering motility and metabolic activation [90,91]. Collectively, these properties suggest that alternative media formulated with molasses and seed extracts can enhance both bacterial growth and chemotactic responses.

Given that the alternative culture medium with soybean seed extract elicited the highest chemotactic response, it was selected to evaluate the shelf-life stability of strain 5P1 in formulations stored at 4 °C. From a biotechnological perspective, the culture medium used during inoculant development should serve not only as a support for achieving high bacterial concentrations but also for ensuring long-term cell survival [92]. In this study, inocula were formulated using fresh culture medium, which is nutrient-rich and free of metabolic by-products that could inhibit cell proliferation, an approach that may allow bacterial multiplication to continue for a longer period during storage. Additionally, storage at low temperature (4 °C) promotes cell survival over extended periods by reducing metabolic activity [24]. Nevertheless, bacterial concentration declined over time across all treatments, most markedly in the unsupplemented control (from 10^10^ to 10^2^ CFU mL^−1^) (Figure 4). Even under these conditions, metabolic activity does not completely cease, leading to the accumulation of physiological damage including oxidative stress, membrane destabilization, and protein and DNA degradation, ultimately resulting in cell death during storage [93]. Previous studies have consistently reported a decline in bacterial viability in rhizobial inoculants during storage, regardless of the carrier used. For instance, *Rhizobium leguminosarum* populations decreased from 10^11^–10^12^ to ≤10^9^ CFU g^−1^ after approximately 6 months of storage [26]. Similarly, liquid biofertilizers have shown reductions from initial concentrations of 10^7^–10^8^ CFU mL^−1^ to 10^4^–10^5^ CFU mL^−1^ over a 5-month period [94]. Even in optimized formulations containing *Bradyrhizobium* spp. or *Rhizobium tropici*, more moderate but continuous decreases of 1–2 log units have been reported over 3–6 months of storage [23,95].

Despite the decline in concentration of *Rhizobium* sp. strain 5P1 over time, cell densities above 10^8^ CFU mL^−1^ were maintained for more than three months when sodium alginate and CMC were used as additives (Figure 4). Culture media supplemented with CMC and sodium alginate have been shown to enhance the shelf-life of PGPB, including *Rhizobium*, *Bradyrhizobium*, *Azospirillum*, and *Pseudomonas* [23,95,96,97]. Both compounds have been widely reported to extend the shelf-life of biofertilizers when used as formulation additives, acting as protective agents by forming a hydrated matrix surrounding the cells, promoting water retention, and reducing desiccation damage. Furthermore, they contribute to membrane stabilization and buffer osmotic fluctuations, thereby reducing the loss of cellular integrity during storage [28,98,99]. These formulations can help maintain high concentrations of rhizobia and other PGPB in inoculants for cereal crops such as rice, a key factor for ensuring effective plant colonization, local production of bioactive metabolites such as IAA, persistence under competition with native soil microbiota, and overall plant growth promotion [23,100]. Therefore, in addition to cell concentration, it is relevant to evaluate the plant growth-promoting (PGP) activity of long-preserved bioproducts to ensure their effectiveness under field conditions.

## 5. Conclusions

This study highlights the potential of *Rhizobium* sp. strain 5P1 and an alternative culture medium as a tool for the development of a promising bioinoculant for rice cv. Selección 1, a variety of high economic relevance in Cuba. Originally isolated from cv. INCA LP-7, strain 5P1 effectively promoted the growth of cv. Selección 1, indicating a low degree of host specificity. The plant growth-promoting traits identified in this strain, including production of indolic compounds and antagonistic activity against important rice fungal phytopathogens, make it an attractive candidate for bioinoculant development. The multiple motility mechanisms and the early activation of plant–bacterium signaling induced by the alternative culture medium may further favor competitive establishment in the rice rhizosphere and enhance the phytostimulatory effect of the strain. Moreover, the alternative culture medium, formulated with molasses and aqueous soybean seed extract and supplemented with CMC or sodium alginate, maintained cell viability above 10^8^ CFU mL^−1^ for more than 3 months at 4 °C, meeting the concentration threshold required for effective field application. Further studies are warranted, including inoculation trials under greenhouse and field conditions, to evaluate the effect of stored inocula on rice growth promotion, as well as compatibility with fungicides and herbicides commonly used in rice production.

## Figures and Tables

**Table 1:** Strains used in this study.

Strain * (GenBank Accession)	Origin	Main Features	Reference
Rpd16 (MT387212) Rpr11 (MT387213)	Rice cv. INCA LP5	The strains produce indolic compounds, siderophores, and biofilms, and solubilize inorganic phosphate. Some strains also exhibit exocellulase activity and antagonistic activity against *Magnaporthe oryzae*, and have been shown to enhance potassium uptake and seed germination in rice and *Coffea* sp.	[46,47,48,49]
5P1 (MT759831)	rice cv. INCA LP7	The strain enhances nitrogen, potassium, chlorophyll, carbohydrate, and protein contents in rice plants. Furthermore, under field conditions, the strain increases tiller number, filled grains per panicle, and grain yield in rice.	[47]
*Magnaporthe oryzae* strain 28, *Curvularia oryzae* strain 1, and *Bipolaris oryzae* strain 1	Rice	Pathogens were isolated from rice plants showing typical disease symptoms and identified morphologically based on mycelium, conidiophore, and conidia characteristics observed on PDA medium under light microscopy.	Micoteca, INCA

* Fractions in parentheses represent the access number of consensus sequences deposited in GenBank.

**Table 2:** Chemical characteristics of the substrate used in the greenhouse inoculation assays of rice cultivar *Selección 1*. The substrate consisted of a 3:1 (*v*/*v*) mixture of Ferric Gleysol soil from Los Palacios, Pinar del Río, Cuba, and organic matter.

pH	OM	P_2_O_5_	Ca^2+^	Mg^2+^	Na^2+^	K^+^
(KCl)	(%)	(mg 100 g^−1^ Soil)	(cmol_c_ kg^−1^)			
6.57 ± 0.27	3.05 ± 0.70	75.1 ± 6.5	11.62 ± 0.97	4.75 ± 0.1	traces	0.80 ± 0.02

Mean values of three composite substrate samples. pH was determined potentiometrically. Organic matter (OM, %) was quantified using the Walkley–Black method. Available P_2_O_5_ (mg 100 g^−1^ substrate) was extracted with 0.1 N H_2_SO_4_. Exchangeable cations were extracted with NH_4_OAc; Ca^2+^ and Mg^2+^ were quantified by complexometric titration, and Na^+^ and K^+^ by flame photometry.

**Table 3:** Effect of culture media on the growth and chemotactic response of *Rhizobium* sp. strain 5P1. The negative controls consisted of Tryptone-Yeast (TY) medium and saline solution for the bacterial growth and chemotaxis assays. Data are presented as means ± standard error of 5 replicates (Tukey’s HSD, *p* ≤ 0.05, *n* = 5).

Treatments	Bacterial Growth (CFU mL^−1^)	Chemotaxis Response (CFU mL^−1^)
Saline solution	-	8.1 × 10^5^ ± 1.2 × 10^5^ c
TY medium	1.7 × 10^10^ ± 7.3 × 10^9^	-
Tym ^a^ medium	1.2 × 10^10^ ± 3.2 × 10^9^	6.1 × 10^5^ ± 1.3 × 10^5^ c
Bf-Sm ^a^ medium	9.5 × 10^9^ ± 4.6 × 10^9^	2.8 × 10^6^ ± 3.8 × 10^5^ a
Ers2V1 ^a^ medium	2.1 × 10^10^ ± 9.2 × 10^8^	1.5 × 10^6^ ± 3.0 × 10^5^ b
Sex	NS	2.6 × 10^5^

^a^ TYm: modified TY medium (pH 5.0, NaCl 15 g L^−1^); Bf-Sm: modified Bradyfact^®^ medium with aqueous soybean seed extract (pH 5.0, NaCl 15 g L^−1^); Ers2V1: modified Bradyfact^®^ medium with aqueous rice seed extract (pH 5.0, NaCl 15 g L^−1^). NS: not significant.

## Data Availability

The original contributions presented in this study are included in the article. Further inquiries can be directed to the corresponding authors.
